# 2692. Epidemiology and Risk Factors for *Clostridioides difficile* Infection after Pancreas Transplantation

**DOI:** 10.1093/ofid/ofad500.2303

**Published:** 2023-11-27

**Authors:** Haris Akhtar, Zachary A Yetmar, Maria T Seville, Wendelyn Bosch, Elena Beam

**Affiliations:** Mayo Clinic Rochester, Rochester, Minnesota; Mayo Clinic, Rochester, Minnesota; Mayo Clinic Arizona, Phoenix, Arizona; Mayo Clinic, Rochester, Minnesota; Mayo Clinic, Rochester, Minnesota

## Abstract

**Background:**

*Clostridioides difficile* infection (CDI) is a common and potentially life-threatening infection. Transplant recipients are vulnerable to CDI due to immune suppression and frequent antibiotic exposure. CDI can lead to serious complications in these patients, including allograft failure. In a population of pancreas transplant recipients, we aimed to identify epidemiologic characteristics and risk factors associated with CDI within 90 days of transplant.

**Methods:**

We performed a retrospective cohort study of adults who underwent pancreas transplantation between January 1, 2010, and December 31, 2020, at three centers in Arizona, Florida, and Minnesota. Patients were excluded if they developed pancreas allograft failure within 48 hours of transplantation. The primary outcome was diagnosis of CDI within 90 days of transplantation and the secondary outcome was pancreas allograft failure. The main analyses were performed by Cox regression.

**Results:**

A total of 482 patients were included, of which 35 (7.3%) developed CDI within 90 days (Table 1). Within the subset of 35 CDI patients, the median time to CDI was 17.0 (IQR 13.0–28.5) days (Figure 1) with most (83%) diagnosed with non-severe CDI. Most patients with CDI were on current systemic antibiotics (94%), primarily for prophylaxis (73%). CDI treatment was vancomycin for the majority of patients (71%) and metronidazole for the rest (29%). One patient had a fecal transplant for recurrence, and none required surgery. In multivariable cox regression of associations with 90-day CDI, significant associations were anastomotic leak (HR 3.04, 95% CI 1.2-7.4; p=0.015) and simultaneous pancreas-kidney transplantation (HR 8.70, 95% CI 1.15-66.7; p=0.036); age (HR 0.97, 95% CI 0.94-1.01; p=0.110) was not significant. In a secondary analysis, CDI was not associated with pancreas allograft failure (p=0.719).

**Table 1.**
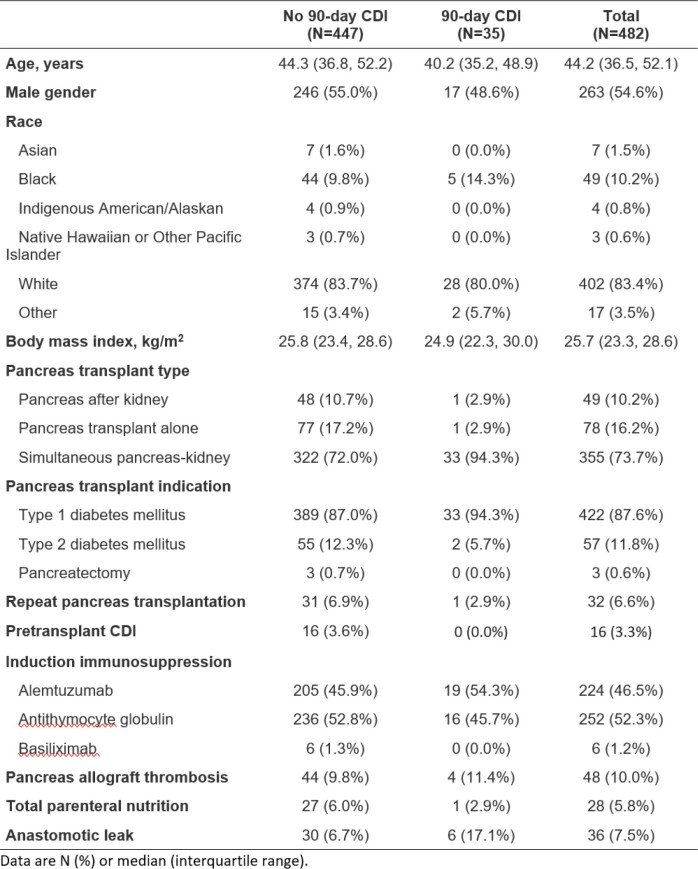
Characteristics of 482 patients who underwent pancreas transplantation.

**Figure:**
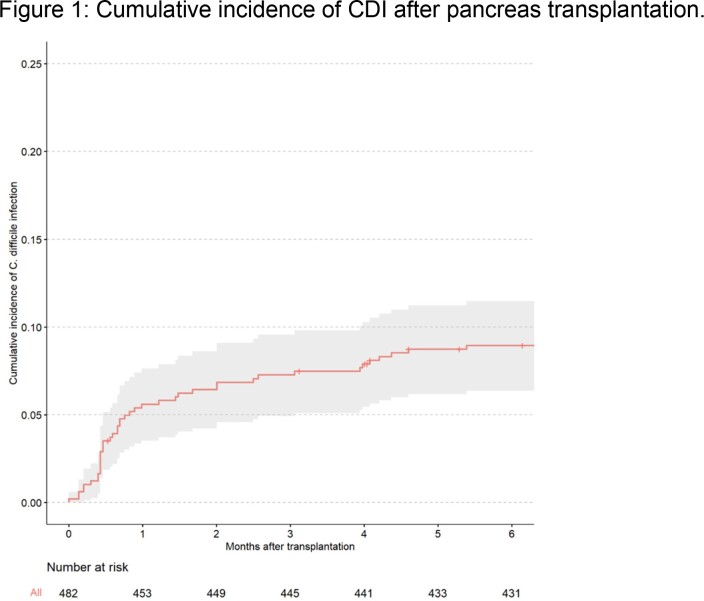

**Conclusion:**

This study identified preceding anastomotic leak as a risk factor for 90-day CDI after pancreas transplantation, likely due to subsequent intra-abdominal infection and antibiotic use. simultaneous pancreas-kidney transplantation is another risk factor due to additional comorbidity. Transplant providers should be cognizant of the risks of CDI and mitigate these risks as possible.

**Disclosures:**

**All Authors**: No reported disclosures

